# Combined Effects of Gas Composition in Modified Atmosphere Packaging and Chitooligosaccharide-EGCG on Quality Changes in Refrigerated Asian Hard Clam Meat

**DOI:** 10.3390/foods15061026

**Published:** 2026-03-15

**Authors:** Ajay Mittal, Claret Shalini D’souza, Mohammad Fikry, Matsapume Detcharoen, Soottawat Benjakul, Feby Luckose, Nurul Huda, Premy Puspitawati Rahayu, Avtar Singh

**Affiliations:** 1 International Center of Excellence in Seafood Science and Innovation (ICE-SSI), Faculty of Agro-Industry, Prince of Songkla University, Hat Yai 90110, Songkhla, Thailand; 2 Department of Food Science, School of Life Sciences, St. Aloysius (Deemed to be University), Mangaluru 575003, Karnataka, India; 3 Date Palm Research Center of Excellence, King Faisal University, Al-Ahsa 31982, Saudi Arabia; 4 Division of Biological Science, Faculty of Science, Prince of Songkla University, Hat Yai 90110, Songkhla, Thailand; 5 Department of Food and Nutrition, Kyung Hee University, Seoul 02447, Republic of Korea; 6 Postgraduate School, Universitas Brawijaya, Malang 65145, East Java, Indonesia; 7 Faculty of Animal Science, Universitas Brawijaya, Malang 65145, East Java, Indonesia

**Keywords:** Asian hard clam, chitooligosaccharide-epigallocatechin gallate, modified atmosphere packaging (MAP), shelf-life extension, refrigerated storage

## Abstract

The influence of different gas compositions in modified atmospheric packaging (MAP) without and with chitooligosaccharide-EGCG (CE) conjugate on storage stability of Asian hard clam (HC) meat during storage at 4 °C was studied. Microbial load of HC meat was <5 log CFU/g when packaged under MAP, regardless of treatment, up to 18 days of storage, whereas control exceeded viable bacterial count (6 log CFU/g) on day 9. The lowest microbial load, volatile bases, and lipid oxidation were obtained in HC meat pretreated with 600 ppm of CE conjugate and MAP (80% CO_2_/20% O_2_) (MAP4-CE) (*p* < 0.05). Correlation heatmap analysis showed that a high-CO_2_/low-O_2_ atmosphere was the primary determinant of reduced *Pseudomonas* growth and lipid oxidation in HC meat, whereas the CE conjugate conferred only minor oxidation and nitrogenous spoilage indices. HC packed under MAP exhibited higher cooking and drip loss, along with increased toughness and firmness, irrespective of treatment. PUFA of MAP4-CE was retained during 18 days of storage. High-CO_2_, with or without CE, redirected the microbial diversity toward CO_2_-tolerant taxa. Overall, MAP4-CE had an extended shelf-life of at least 18 days while better preserving lipid quality and delayed growth of spoilage bacteria.

## 1. Introduction

Asian hard clam (HC; *Meretrix lusoria*) is widely consumed due to its nutritional quality and affordability in Thailand. However, high water activity and protein content and neutral pH of HC make it an ideal substrate for bacterial proliferation, which limits the shelf-life and presents a challenge for the seafood industry. HC is available as raw, unshelled or shucked, chilled or frozen products in vacuum-sealed bags in the market. The storage at lower temperature is applied to extend the shelf-life of the bivalve to 4–5 days, depending upon initial microbial load and diversity. Frozen storage of seafood has been known to terminate microbial growth, impede enzyme activity, and reduce lipid oxidation and protein denaturation [1,2]. However, long-term frozen storage contributes to deterioration in flavor, texture, and color and increased exudates [3]. Modern consumers are seeking fresh seafood rather than frozen or processed seafood, which is possibly satisfied through the application of modified atmosphere packaging (MAP).

MAP is an effective preservation technology to maintain the quality and extend the shelf-life of seafood. Moreover, MAP at lower temperatures has proved to be an effective preservation method for shelf-life extension and quality retention of various food products. MAP has been successfully used for shelf-life extension of red drum fillets, salmon, whiting fillets, and other seafood [4,5,6,7,8]. In MAP, seafood is packed with different gases, mainly CO_2_, O_2_, and N_2,_ at different ratios to inhibit bacterial growth, reduce proteolysis, and lower lipid oxidation. CO_2_ has bacteriostatic activity by extending the lag phase of aerobic bacteria [9]. In addition, O_2_ from the package is replaced by inert N_2_, which suppresses aerobic microorganism growth, decreases oxidative rancidity, and prevents packaging collapse, while O_2_ reduces drip loss and maintains meat color [10,11,12]. Nevertheless, proteins and lipids are susceptible to oxidation in the presence of O_2_. Raised CO_2_ levels from 20% to 60% were generally required in MAP to strengthen protection against aerobic spoilage organisms [13]. CO_2_ had an influence on meat quality, such as texture, color and lipid oxidation, which has been less researched and usually ignored, and was even controversial. It has been widely reported that atmospheres enriched in CO_2_ could effectively suppress meat lipid oxidation [14]. However, in contrast, Esmer et al. [15] found that high levels of CO_2_ favored lipid oxidation with 30% O_2_ or 50% O_2_ in minced beef package. Therefore, gas composition in MAP plays an important role in regulating seafood quality. To optimize preservation, it is essential to determine how different gas compositions affect the physicochemical and microbiological qualities of seafood. Moreover, the negative effects of O_2_ can be reduced by incorporating natural antioxidants before MAP, providing an effective approach to enhance product shelf-life and overall quality.

Chitooligosaccharide (COS) derived from shrimp shell waste is a value-added product that contributes to the principles of circular economy and environmental sustainability. COS can be utilized for the shelf-life extension of seafood due to its antimicrobial and antioxidant activities [16]. Moreover, COS conjugation with epigallocatechin gallate (EGCG)(CE conjugate) has been shown to further enhance its bioactivities and can impede oxidation and microbial spoilage of seafood during refrigerated storage [17,18]. CE conjugate showed antioxidant activities in different systems [19]. These findings highlighted the potential of CE conjugate as a multifunctional natural preservative and its synergistic potential when combined with non-thermal processing technologies. CE conjugate in combination with MAP could enhance oxidative stability and extend the shelf-life of seafood. So far, no study has been reported on the combined impact of MAP and CE conjugate on seafood, especially HC meat. Furthermore, the prevalence of different microbes under various gas compositions in MAP could provide additional information, which could be useful in improving the preservation of HC meat.

Therefore, the objective of the present study was to evaluate the combined effects of CE conjugate and MAP at different concentrations of O_2_/CO_2_/N_2_, particularly of O_2_ and N_2_ under high CO_2_ conditions, on the microbial counts and chemical quality indices, including volatile bases and lipid oxidation stability of HC meat during refrigerated storage. Physical attributes such as drip loss and cooking loss were also monitored during storage. In addition, changes within the microbial community of HC meat during refrigerated storage were determined to evaluate the effect of different gas compositions.

## 2. Materials and Methods

### 2.1. Chemicals and Microbial Culture Media

All chemicals used were of analytical grade. Acetone (CAS 67-64-1, 99.5%), hydrochloric acid (CAS 7647-01-0, 37%), trichloroacetic acid (CAS 76-03-9, 99%), sodium acetate (CAS 127-09-3, 99%), chloroform (CAS 67-66-3, 99.8%), methanol (CAS67-56-1, 99.9%), sodium chloride (CAS 7647-14-5, 99.8%), ammonium thiocyanate (CAS 1762-95-4, 99%), sodium hydroxide (CAS 1310-73-2, 98%), iron chloride (CAS 10025-77-1, 97%), malonaldehyde (CAS 122-31-6, 97%), and cumene hydroperoxide (CAS 80-15-9, 80%) were purchased from Merck (Damstadt, Germany) and Sigma-Aldrich (St. Louis, MO, USA).

Microbial media including plate count agar, *Pseudomonas* agar base, triple sugar iron agar, thiosulfate citrate bile salts sucrose agar, and cephaloridine-fucidin-cetrimide were bought from Oxoid (Thermo Fisher Scientific, Waltham, MA, USA). Polystyrene tray (8.5 cm × 14.5 cm × 1 cm) was purchased from the local market (J.T. Pack of Foods Co., Ltd., Nonthaburi, Thailand). Polyamide/polyethylene (PA/PE-35/35) bags (12.5 cm × 20 cm; thickness: 70 µm) were also purchased from a local supermarket (Manee Udomsuk Co., Ltd., Prawet, Bangkok, Thailand).

### 2.2. Collection and Extraction of Edible Portions from Hard Clam (HC)

Freshly harvested HCs were purchased from a seafood market in Songkhla Province, Thailand, and delivered to the laboratory under chilled conditions within 1 h. The shells were cleaned by brushing, thoroughly rinsed with tap water, and left to drain. The HCs were then vacuum sealed in polyethylene/polyamide bags and blanched by immersion in boiling water for 1 min, followed by immediate cooling in ice water. After cooling, the edible portions were carefully removed by hand using a sterilized knife.

### 2.3. Preparation of CE Conjugate and HC Meat Treatment

CE conjugate was prepared via the H_2_O_2_ and ascorbic acid (AsA) redox pair method as per our previous report [19]. Firstly, COS solution (1%, *w*/*v*) was prepared in distilled water and adjusted to pH 5.0 using 1 M acetic acid. Simultaneously, 1 M H_2_O_2_ (4 mL) containing 0.10 g of AsA was incubated at 40 °C for 15 min to generate hydroxyl radicals. These two solutions were then combined and incubated at room temperature for 1 h with continuous stirring. Following this, EGCG (0.1%, *w*/*w* of COS) was added to the mixture, which was then incubated at room temperature for 24 h in the dark. The resulting mixture was dialyzed using cellulose membrane of 0.5 kDa cutoff to remove unconjugated EGCG, and the dialysate was freeze-dried to obtain CE conjugate powder. CE conjugate had conjugation efficiency of 30% as assayed by the procedure of Mittal, Singh, Zhang, Visessanguan and Benjakul [19]. However, CE conjugate is not approved by the FDA, EFSA, or other regulatory bodies for direct food or food-contact use.

CE conjugate (600 mg) was dissolved in a minimal amount of distilled water for 1000 g of HC meat (final concentration 600 ppm based on preliminary study). Thereafter, HC meat was evenly coated with CE conjugate via application with a brush. The treated HC meat was divided into 100 g batches, placed on a polystyrene tray in polyamide/polyethylene bags (oxygen permeability: 47.62 cm^3^/m^2^/day at 38 °C), packed with a sample-to-gas ratio of 1:3 (*w*/*v*) using a gas mixer (Model KM 20–200-3 ME, WITT Co. Ltd., Nonthaburi, Thailand), and finally sealed. Gas compositions containing 40% CO_2_/20% O_2_/40% N_2_ (MAP1-CE), 60% CO_2_/10% O_2_/30% N_2_ (MAP2-CE), 80% CO_2_/20% N_2_ (MAP3-CE), and 80% CO_2_/20% O_2_ (MAP4-CE) were used. The samples without CE conjugate treatment followed by MAP at the aforementioned gas compositions were named MAP1, MAP2, MAP3, and MAP4, respectively. HC meat treated without and with CE conjugate at 600 ppm was also packed with ambient air. The samples were named CON and CON-CE, respectively. All samples were stored for 18 days at 4 °C and were monitored for physicochemical and microbiological changes at 3-day intervals.

### 2.4. Microbial, Chemical, and Physical Analyses

#### 2.4.1. Microbial Counts

Twenty-five grams of HC meat was blended with 0.8% (*w*/*v*) NaCl solution, and the resulting homogenate was used for microbiological analysis. Total viable bacterial count (TVBC) was determined by the spread plate technique on plate count agar, following the Aerobic Plate Count procedure described in the FDA Bacteriological Analytical Manual Method 13. Psychrophilic bacteria (PBC) were enumerated in accordance with ISO 17410. Presumptive *Pseudomonas* count (PPC) was quantified on *Pseudomonas* agar base supplemented with cephaloridine-fucidin-cetrimide (CFC) selective agents as outlined in ISO 13720. H_2_S-producing bacteria and *Vibrio* spp. were assessed on triple sugar iron (TSI) agar and thiosulfate citrate bile salts sucrose (TCBS) agar, respectively. Plates for psychrophilic counts were incubated at 4 °C for 7–10 days, whereas all other cultures were incubated at 37 °C for 18–24 h. Results were reported as log CFU/g of sample.

#### 2.4.2. Volatile Bases and Lipid Oxidation 

Total volatile base-nitrogen (TVB-N) and trimethylamine-nitrogen (TMA-N) contents were determined using the Conway disc method as described by Mittal et al. [20]. TVB-N and TMA-N contents were expressed as mg N/100 g sample. Peroxide value (PV) and thiobarbituric acid reactive substances (TBARS) content were determined for lipid oxidation as suggested by Richards and Hultin [21] and [22], respectively. PV and TBARS content were expressed as mg cumene hydroperoxide/kg sample and mg malonaldehyde (MDA)/kg sample, respectively.

#### 2.4.3. Drip Loss and Cooking Loss

The initial weight of the sample on day 0 (*W_i_*) and final weight of the sample on a particular storage day (*W_d_*) after treatment as described in Section 2.3 were measured using an analytical balance and used to calculate the drip loss according to the following equation:

$$Drip\;loss\;\left(\%\right)=\left(\frac{{W_{i}-W}_{d}}{W_{i}}\right)\times 100$$

Cooking loss was determined by evaluating the weight difference of the samples before and after thermal processing. HC meat was steamed until the core temperature reached 85 °C. The samples were cooled after steaming, and excess surface moisture was gently removed. The initial weight before cooking (*A*) and final weight after cooking (*B*) were recorded. Cooking loss was then calculated using the following equation:

$$Cooking\;loss\;\left(\%\right)=\frac{\left[A-B\right]}{A}\times 100$$

#### 2.4.4. Textural Analysis

Textural properties of HC meat were assessed using a TA-XT2 texture analyzer (Stable Micro Systems, Surrey, UK) equipped with a Warner–Bratzler blade at a speed rate of 2 mm/s and a 50 kg load cell. The blade was applied perpendicularly to the axis of muscle fibers. Firmness and toughness were recorded to assess structural integrity and resistance to deformation in the samples. Ten samples per treatment were evaluated for each texture parameter.

#### 2.4.5. Fatty Acids Profile

All samples on day 0 and samples that exhibited the lowest microbial load at the end of storage (MAP4 and MAP4-CE on day 18) were selected for fatty acid profile analysis. Lipid from HC meat was extracted using the Bligh and Dyer method [23]. Subsequently, lipid transmethylation was performed using 2 M methanolic NaOH and 2 M methanolic HCl to yield fatty acid methyl esters (FAMEs). FAMEs were separated using an Agilent 7890B (Santa Clara, CA, USA) gas chromatography (GC) system equipped with a flame ionization detector (FID), as suggested by Raju and Benjakul [24]. Individual FAMEs were identified by comparing their retention times to FAME standards. The results were expressed as mg/100 mg lipid.

### 2.5. Correlation Between Treatments and Chemical/Microbial Parameters

To examine how gas composition influenced microbial growth behavior and chemical spoilage indices, a correlation analysis was performed using treatment-level data. For each treatment (CON, CON-CE, MAP1, MAP1-CE, MAP2, MAP2-CE, MAP3, MAP3-CE, MAP4, MAP4-CE), the following variables were compiled in a single data matrix: gas composition (CO_2_ and O_2_ contents and presence of CE conjugate coded as 0 = absent and 1 = present), maximum growth rates of TVBC_µ, PBC_µ and PPC_µ, and chemical indices determined at the selected days of acceptance limit of TVBC (PV, TBARS content, TVB-N and TMA-N). Each treatment was therefore represented by one row in the data set (*n* = 10 observations). Pearson product–moment correlation coefficients (r) and two-tailed *p*-values were calculated in GraphPad Prism 10 (GraphPad Software, San Diego, CA, USA), with *p* < 0.05 considered significant. The resulting correlation matrix was visualized as a heat map, with cell color representing r values from −1 to +1, to highlight clusters of variables responding similarly to changes in gas composition. *Vibrio* spp., LAB, and HSPB were not observed for most of the treated samples; thus, they were not selected for analysis.

### 2.6. Microbial Diversity

Microbial community analysis by 16s rRNA sequencing was carried out at selected storage points showing the highest viable bacterial counts: day 6 for CON, day 12 for CON-CE, and day 18 for all treatments. Genomic DNA was extracted from these samples using a commercial kit (ZymoBIOMICS^®^ DNA Miniprep Kit; Zymo Research, Irvine, CA, USA), following the manufacturer’s instructions. The hypervariable V3–V4 region of the 16S rRNA gene was amplified by PCR with universal bacterial primers, and a defined mock community (ZymoBIOMICS^®^ Microbial Community DNA Standard; Zymo Research) was processed in parallel as a positive control for library preparation. The resulting amplicon libraries were sequenced on an Illumina^®^ NextSeq 2000™(Illumina, San Diego, CA, USA) using a p1 reagent kit (2 × 300 bp, 600 cycles) with a 25% PhiX spike-in. Sequence data were processed in QIIME v.1.9.1, where UCLUST was used for OTU clustering and taxonomic assignment against the curated Zymo Research 16S reference database. Community composition profiles and diversity metrics (alpha and beta diversity) were also generated in QIIME [25].

### 2.7. Statistical Analysis

All trials were carried out under a completely randomized design (CRD), with three independent replicates for each treatment. The resulting data were subjected to one-way analysis of variance (ANOVA). Prior to ANOVA, assumptions of normality and homogeneity of variance were verified using the Shapiro–Wilk test and Levene’s test, respectively (*p* > 0.05). When the ANOVA indicated a significant treatment effect, Tukey’s multiple range test was used to compare means. The value of *p* < 0.05 was considered to indicate statistical significance. For microbial community analysis, beta diversity was estimated using Bray–Curtis dissimilarity. Community patterns were visualized by principal coordinates analysis (PCoA) and non-metric multidimensional scaling (NMDS), using the ordinate function in the phyloseq package v1.54.0. The NMDS goodness-of-fit was evaluated based on stress value. Differences in community composition among treatments were assessed using the vegan package v. 2.7-2 for permutational multivariate analysis of variance (PERMANOVA) with the adonis2 function, and analysis of similarities (ANOSIM).

## 3. Results and Discussion

### 3.1. Changes in Microbial Load of HC Meat

**Figure 1:**
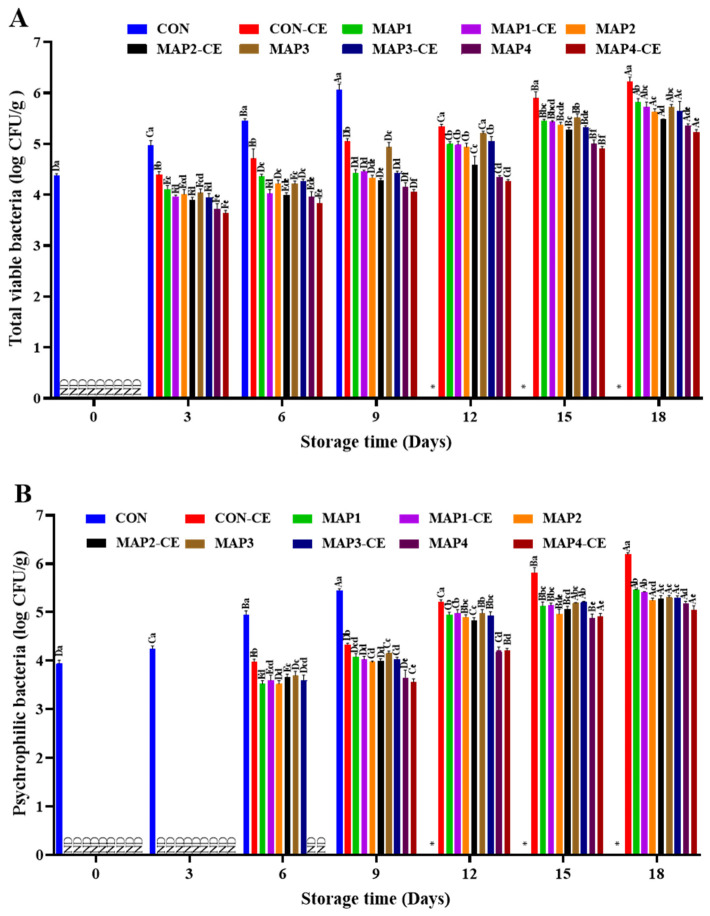
Total viable bacteria (**A**) and psychrophilic bacteria (**B**) counts of hard clam meat treated under various conditions. The error bars represent standard deviation (*n* = 3). Different lowercase letters on the bars denote significant differences between different treatments on the same storage day (*p* < 0.05). Different uppercase letters on the bars denote significant differences between the same treatments on different storage days (*p* < 0.05). *: not determined; ND: not detected; CON: hard clam meat (HC) without any treatment; CON-CE: HC meat treated with chitooligosaccharide-epigallocatechin gallate (CE) conjugate; MAP1: HC meat packed with 40% CO_2_/20% O_2_/40% N_2_; MAP_2_: HC meat packed with 60% CO_2_/10% O_2_/30% N_2_; MAP3: HC meat packed with 80% CO_2_/20% N_2_; MAP4: HC meat packed with 80% CO_2_/20% O_2_. The samples treated with CE conjugate followed by MAP at the aforementioned gas compositions were named MAP1-CE, MAP2-CE, MAP3-CE, and MAP4-CE, respectively.

TVBC for all samples treated under various conditions are given in Figure 1A. On day 0, CON had a TVBC of 4.37 log CFU/g, whereas no detectable bacterial growth was observed in the treated samples. The treatment with either CE conjugate or MAP or a combination of both effectively inhibited bacterial proliferation. TVBC is associated with mesophilic bacterial growth at 20 °C to 45 °C. Aerobic bacteria such as *V. parahaemolyticus*, *Aeromonas*, and *Enterobacter* are present in seafood and can contribute to spoilage and potential foodborne illnesses [26]. TVBC increased in all samples as storage time progressed. On day 3, TVBC reached 4.96 log CFU/g in CON. Among treated samples, CON-CE showed the highest TVBC; however, the value was within the acceptable limits on all storage days except day 18 (*p* < 0.05). The result suggested that the CE conjugate showed antibacterial activity due to its ability to disrupt biofilm formation, impair bacterial motility, and induce cell wall damage, ultimately leading to protein leakage and DNA binding. HC meat treated with CE conjugate in combination with MAP had lower TVBC than that treated with MAP alone, irrespective of the gas composition. MAP1-CE exhibited lower TVBCs than MAP1 (*p* < 0.05), while the other remaining MAP-treated samples did not vary from their respective counterparts (*p* > 0.05). MAP1-CE and MAP2-CE showed significantly lower TVBCs than MAP1 and MAP2, respectively, on day 6 as well (*p* < 0.05). In contrast, no significant differences were observed between MAP3 and MAP3-CE or MAP4 and MAP4-CE on day 6 (*p* > 0.05). These findings indicated that the CE conjugate acted synergistically with MAP to enhance bacterial inactivation. When a higher proportion of CO_2_ was used, lower TVBC values were obtained. For instance, MAP4 and MAP4-CE exhibited the lowest TVBC (*p* < 0.05), and the difference between them was not significant on day 9 (*p* > 0.05). This finding can be attributed to the bacteriostatic effect of CO_2_ as it dissolves with free water in meat and forms carbonic acid (H_2_CO_3_) that lowers the pH and disrupts bacterial cell membranes [27]. This leads to reduced enzyme activity and metabolic inhibition, thereby slowing bacterial growth. CON showed the most significant increase in TVBC, surpassing the acceptable limit for seafood consumption (6 log CFU/g) on day 9 (*p* < 0.05) [28]. In contrast, treated samples maintained lower TVBCs (4.05 to 5.33 log CFU/g). Moreover, MAP3 and MAP3-CE had higher TVBCs than MAP4 and MAP4-CE, respectively, on day 12 (*p* < 0.05). The presence of O_2_ might enhance the inhibitory effect of CO_2_ by imposing oxidative stress on spoilage bacteria, which are facultative anaerobes or microaerophilic. Overall, results indicate that the gas composition had a crucial role in microbial inhibition during refrigerated storage.

Changes in the PBC of samples during refrigerated storage are presented in Figure 1B. The initial PBC of all treated samples was below the detectable limit, while the CON showed a count of 3.93 and 4.25 log CFU/g on days 0 and 3, respectively. During storage, a gradual increase in PBC was observed in all treatments; however, the rate of increase varied depending on the packaging atmosphere and the presence of CE conjugate. On day 6, the PBC in the CON reached 4.95 log CFU/g, whereas the treated samples remained between 3.52 and 3.98 log CFU/g. MAP4 and MAP4-CE had no detectable growth of psychrophiles on day 6. At the onset of refrigerated storage, psychrophilic bacteria were not dominant; however, these bacteria rapidly grew and became predominant as the storage period progressed. As storage progressed to days 9 and 12, a marked increase in PBC was observed, with the CON exceeding 6 log CFU/g. Among the MAP-treated samples, those containing CE conjugate exhibited PBCs similar to those of samples without CE conjugate (*p* > 0.05). Among these, treatments containing a higher CO_2_ proportion (MAP4 and MAP4-CE) consistently exhibited the lowest PBCs. In particular, on day 12, MAP3 and MAP3-CE (CO_2_/N_2_ atmosphere) showed higher PBCs than MAP4 and MAP4-CE (CO_2_/O_2_ atmosphere) (*p* < 0.05). This could be associated with the inert nature of N_2_; on the other hand, the higher antibacterial activity under CO_2_ might have surpassed the effect of O_2_ as a favorable gas for aerobic bacteria. Psychrotrophic bacteria, such as *Shewanella*, *Pseudomonas*, and *Photobacterium*, are generally responsible for spoilage in seafood during cold storage [29].

**Figure 2:**
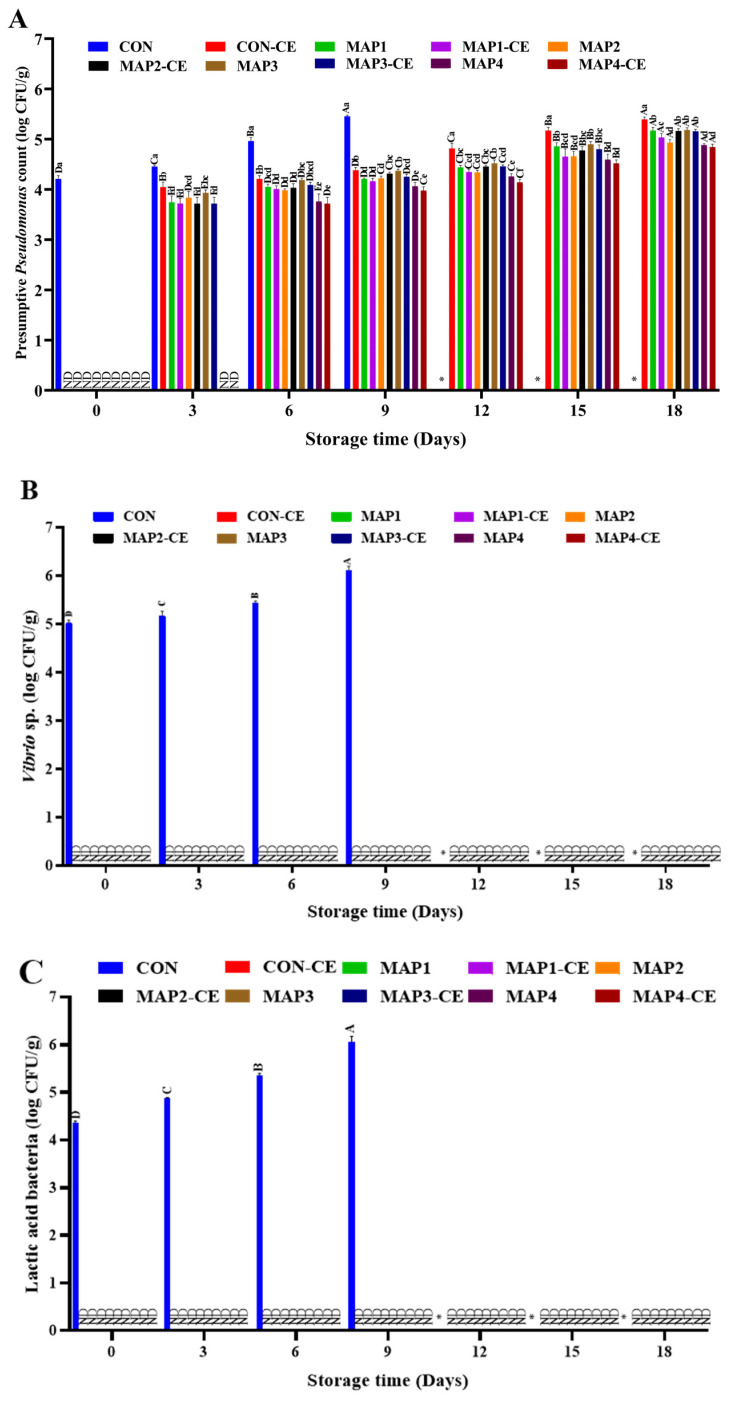

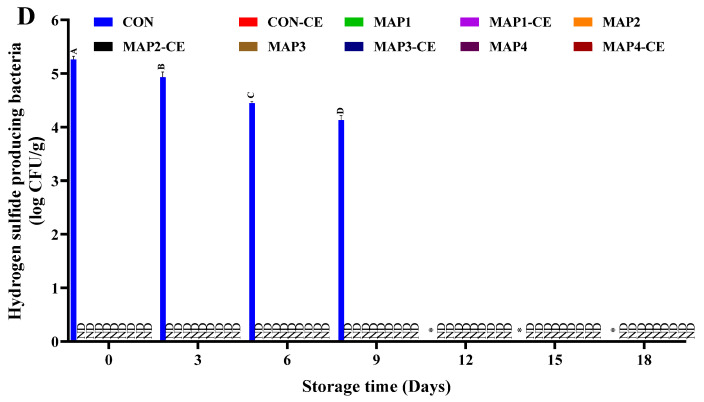
Presumptive *Pseudomonas* spp. (**A**), *Vibrio* sp. (**B**), lactic acid bacteria (**C**), and hydrogen sulfide-producing bacteria (**D**) counts of hard clam meat treated under various conditions. The error bars represent standard deviation (*n* = 3). Different lowercase letters on the bars denote significant differences between different treatments on the same storage day (*p* < 0.05). Different uppercase letters on the bars denote significant differences between the same treatments on different storage days (*p* < 0.05). *: not determined; ND: not detected; CON: Hard clam meat (HC) without any treatment; CON-CE: HC meat treated with chitooligosaccharide-epigallocatechin gallate (CE) conjugate; MAP1: HC meat packed with 40% CO_2_/20% O_2_/40% N_2_; MAP_2_: HC meat packed with 60% CO_2_/10% O_2_/30% N_2_; MAP3: HC meat packed with 80% CO_2_/20% N_2_; MAP4: HC meat packed with 80% CO_2_/20% O_2_. The samples treated with CE conjugate followed by MAP at the aforementioned gas compositions were named MAP1-CE, MAP2-CE, MAP3-CE, and MAP4-CE, respectively.

The changes in PPC of various treatments during refrigerated storage are presented in Figure 2A. On day 0, all treated samples showed no detectable *Pseudomonas*, whereas the CON exhibited an initial load of 4.21 log CFU/g. By day 3, a significant increase (*p* < 0.05) in PPC was observed in the CON, reaching about 4.46 log CFU/g sample, whereas the treated samples showed markedly lower counts (3.72–4.05 log CFU/g sample). MAP3-CE and MAP4 had no detectable growth of *Pseudomonas* on day 3 as well. On day 6, all treatments continued to show gradual increases in PPC, though significantly (*p* < 0.05) lower than that of the CON (4.96 log CFU/g sample). The samples treated with high CO_2_ (80% with 20% O_2_), particularly MAP4 and MAP4-CE, had PPCs of 3.76 and 3.72 log CFU/g, whereas samples showed relatively higher growth, specifically when O_2_ was replaced with N_2_ (MAP3 and MAP3-CE), irrespective of CE conjugate addition. By day 9, microbial proliferation became more pronounced in all treatments, yet the CON reached 5.56 log CFU/g, significantly exceeding the treated groups. Treatments with 80% CO_2_ with 20% O_2_ (MAP4 and MAP4-CE) remained most effective, while other treatments consistently exhibited PPCs around 4.2 log CFU/g, except for CON-CE (4.38 log CFU/g). The combined effect of elevated CO_2_ and CE conjugate appeared to exert a bacteriostatic influence, delaying microbial spoilage. After 12 days of storage, the CON recorded the highest PPC (5.92 log CFU/g), while CON-CE had a PPC of 4.82 CFU/g, and all MAP-treated samples maintained significantly lower levels (4.14–4.52 log CFU/g), irrespective of CE conjugate addition. VC, LABC, and HSPBC are given in Figure 2B, Figure 2C and Figure 2D, respectively. Among all samples, irrespective of treatment, only CON exhibited *Vibrio*, lactic acid bacteria, and H_2_S-producing bacteria. On day 0, CON had VC, LABC, and HSPBC of 5.01, 4.36, and 5.26 log CFU/g. Similarly to other bacterial counts, the abundance of these bacteria increased with prolonged storage duration (*p* < 0.05). Overall, these results demonstrate that the CE conjugate acted synergistically with MAP to enhance bacterial inactivation, and that both CO_2_ concentration and choice of carrier gas play crucial roles in controlling microbial stability during storage.

### 3.2. Changes in TVB-N and TMA-N Contents

**Figure 3:**
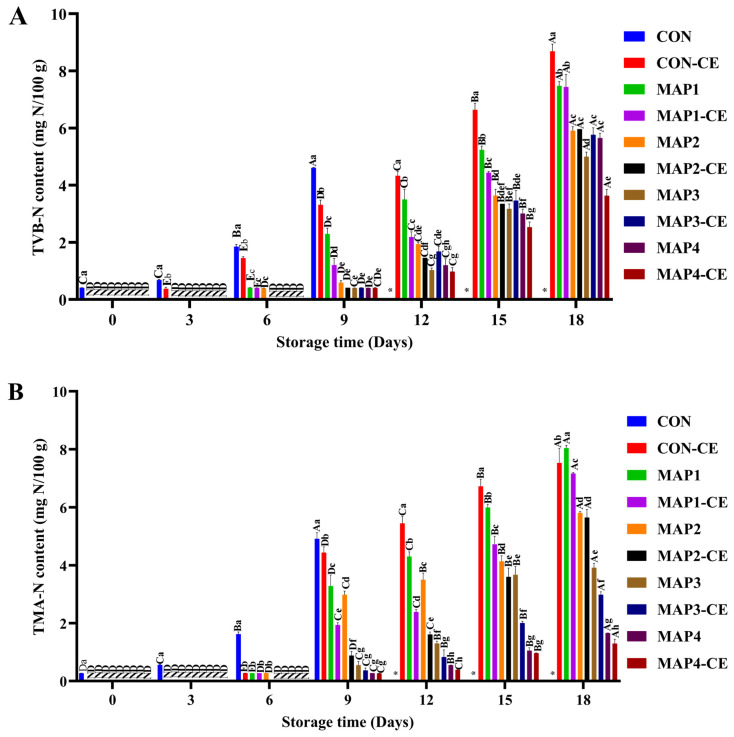

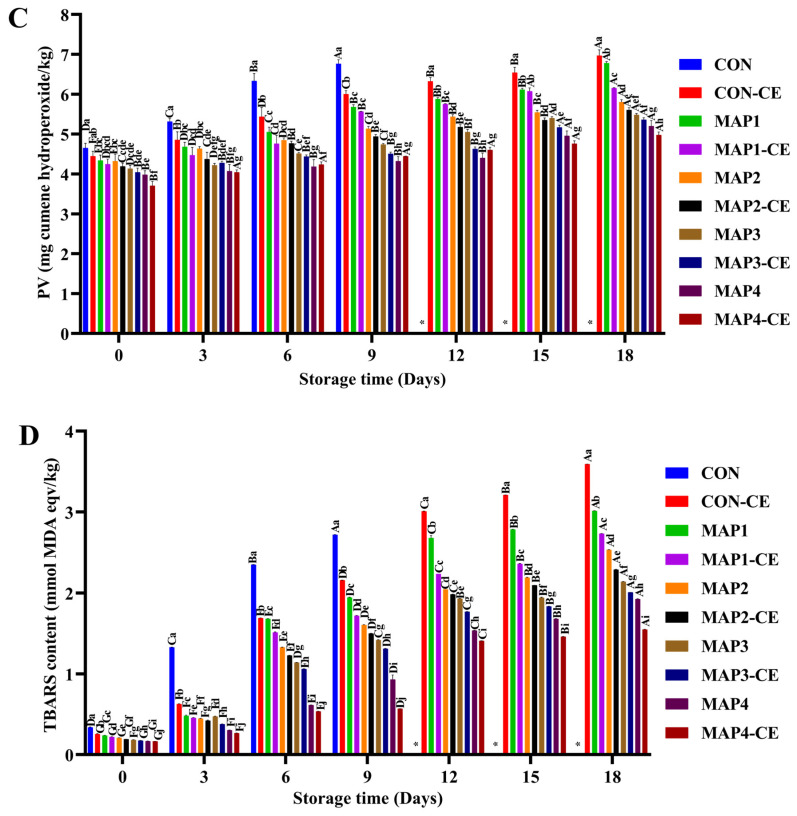
Total volatile nitrogen base content (**A**), trimethylamine content (**B**), peroxide value (**C**), and thiobarbituric acid reactive substances content (**D**) of hard clam meat treated under various conditions. The error bars represent standard deviation (*n* = 3). Different lowercase letters on the bars denote significant differences between different treatments on the same storage day (*p* < 0.05). Different uppercase letters on the bars denote significant differences between the same treatments on different storage days (*p* < 0.05). *: not determined; ND: not detected; CON: Hard clam meat (HC) without any treatment; CON-CE: HC meat treated with chitooligosaccharide-epigallocatechin gallate (CE) conjugate; MAP1: HC meat packed with 40% CO_2_/20% O_2_/40% N_2_; MAP_2_: HC meat packed with 60% CO_2_/10% O_2_/30% N_2_; MAP3: HC meat packed with 80% CO_2_/20% N_2_; MAP4: HC meat packed with 80% CO_2_/20% O_2_. The samples treated with CE conjugate followed by MAP at the aforementioned gas compositions were named MAP1-CE, MAP2-CE, MAP3-CE, and MAP4-CE, respectively.

Generally, TVB-N and TMA-N contents indicate the quality and freshness of seafood [30]. TVB-N contents of all samples during refrigerated storage are given in Figure 3A. TVB-N was not detected in any samples except CON (0.41 mg N/100 g) on day 0. As storage progressed to day 3, the TVB-N content in the CON increased to 0.65 mg N/100 g. In comparison, CON-CE showed a TVB-N content of 0.38 mg N/100 g, while no detectable TVB-N was observed in MAP-treated samples, irrespective of gas composition and CE addition. The lower TVB-N content in CON-CE, as compared to CON, indicated CE helped to retard spoilage by inhibiting oxidative reactions and indirectly suppressing microbial activity. This trend persisted throughout the entire storage period. The data was also supported by the lower TVBC (Figure 1A). TVB-N content gradually increased in all samples as storage time increased. On day 6, CON had the highest TVB-N content, followed by CON-CE, MAP2, MAP1, and MAP1-CE, respectively. However, there were no differences between MAP2, MAP2-CE, and MAP3 (*p* > 0.05), although all showed lower TVB-N content than CON-CE (*p* < 0.05). These results indicate that MAP, in the presence of CO_2_, especially at a higher proportion, effectively reduced the TVB-N content, suggesting its antimicrobial activity. Upon further storage extension to day 9, the TVB-N values of MAP2-CE, MAP3, MAP3-CE, MAP4, and MAP4-CE were similar (*p* > 0.05), and all were significantly lower (*p* < 0.05) than those of the other samples on the same day. The lower TVB-N content observed in MAP2-CE compared to MAP2 (*p* < 0.05) was attributed to the antioxidant and antimicrobial properties of the CE conjugate. In contrast, the reduced TVB-N levels in MAP3, MAP3-CE, MAP4, and MAP4-CE may have been due to the high proportion of CO_2_ in these treatments, which exert antimicrobial effects. The changes in TVB-N content among all treatments became more pronounced after day 12. The addition of CE conjugate effectively reduced TVB-N content, irrespective of gas composition.

TMA was not detected in any of the treatments other than CON up to 3 days of storage (Figure 3B). As storage progressed to day 6, a slight increase in TMA was observed, especially for CON-CE, MAP1, MAP1-CE, and MAP2, but levels remained low (<1 mg N/100 g sample), suggesting the early onset of microbial activity. TMAO reductase from spoilage bacteria catalyzes the reduction of TMAO to TMA, which contributes to off-odors and flavors [31]. From day 9 onward, TMA accumulation became more pronounced, with the CON showing the highest TMA content (4.91 mg N/100 g) at day 9, followed by CON-CE (4.44 mg N/100 g) and MAP1 (3.29 mg N/100 g), while other treatments exhibited significantly lower levels, highlighting a stronger inhibitory effect on spoilage. HSPB, particularly *Shewanella* spp., catalyzes the reduction of TMAO to TMA through the action of TMAO reductase, which is responsible for the characteristic fishy odor in refrigerated seafood. Accordingly, TVB-N and TMA-N levels were closely associated with the extent of microbial growth and were influenced by the specific preservation treatments applied.

### 3.3. PV and TBARS Content

PV and TBARS values for CON and the treated samples are presented in Figure 3C and Figure 3D, respectively. On day 0, PV ranged from 3.71 to 4.65 mg cumene hydroperoxide equivalent/kg across all treatments. During chilled storage, PV gradually rose in every group, with CON consistently exhibiting the greatest increase (*p* < 0.05). Since PV reflects the accumulation of hydroperoxides, it serves as an indicator of primary lipid oxidation in HC meat. The use of MAP slowed lipid oxidation compared with the untreated control, resulting in lower PV values in the MAP-treated samples. An increase in CO_2_ tended to give lower PV, possibly due to limited microbial growth and the release of a lipolytic enzyme [32]. The lowest PV was obtained when HC meat was packed in 80% CO_2_ and 20% O_2_. Moreover, when the CE conjugate was added before MAP, HC meat exhibited lower PV than in samples treated only with MAP. CE conjugate possessing NH_2_ and OH groups could scavenge free radicals and had the potential to transfer H atoms to free radicals [33].

The highest and lowest TBARS content were in CON and MAP4-CE on day 0, respectively (*p* < 0.05). Like PV, the TBARS content of all samples increased as the storage period increased. It was increased due to the formation of various secondary oxidation compounds, such as ketones and aldehydes, corresponding to unpalatable levels of rancid flavor or odors [34]. The highest TBARS level was observed for CON on day 9 (*p* < 0.05). Like PV, TBARS levels were lower in HC meat treated with CE conjugate in combination with MAP, regardless of gas composition. Moreover, HC meat packed in 80% CO_2_ and 20% O_2_ possessed lower TBARS content than samples packed in other treatments, irrespective of CE conjugate incorporation. Notably, when O_2_ was replaced with N_2_ (i.e., 80% CO_2_ and 20% N_2_), higher TBARS values were obtained (*p* < 0.05). The absence of oxygen suppresses endogenous antioxidant enzyme systems, which depend on oxygen or a balanced redox potential for activity [35]. Consequently, pro-oxidant factors accumulate, while enzymatic antioxidant defense mechanisms are diminished, resulting in a higher rate of lipid oxidation despite the absence of oxygen. Additionally, differences in microbial communities under O_2_- and N_2_-containing atmospheres could affect lipid oxidation processes through microbial metabolism and enzyme production. Treatments held under atmospheres containing 60–80% CO_2_ exhibited higher TBARS values than those stored with lower CO_2_ levels. This may be attributed to the formation of carbonic acid when CO_2_ dissolves in the muscle, which can promote protein denaturation and thereby release heme-bound iron, a strong pro-oxidant in muscle systems [36,37]. At elevated CO_2_ concentrations, the greater carbonic acid load in the tissue may further impair endogenous antioxidant enzymes, leading to an overall increase in lipid oxidation [38]. In the present study, MAP4-CE exhibited the lowest TBARS value (1.55 mg MDA/kg) after 18 days of storage, remaining below this threshold throughout the storage period. Generally, TBARS content up to 2 mg MDA/kg of seafood is regarded as the threshold beyond which undesirable odors or flavors are developed [39].

### 3.4. Changes in the Physical Characteristics of HC Meat

**Figure 4:**
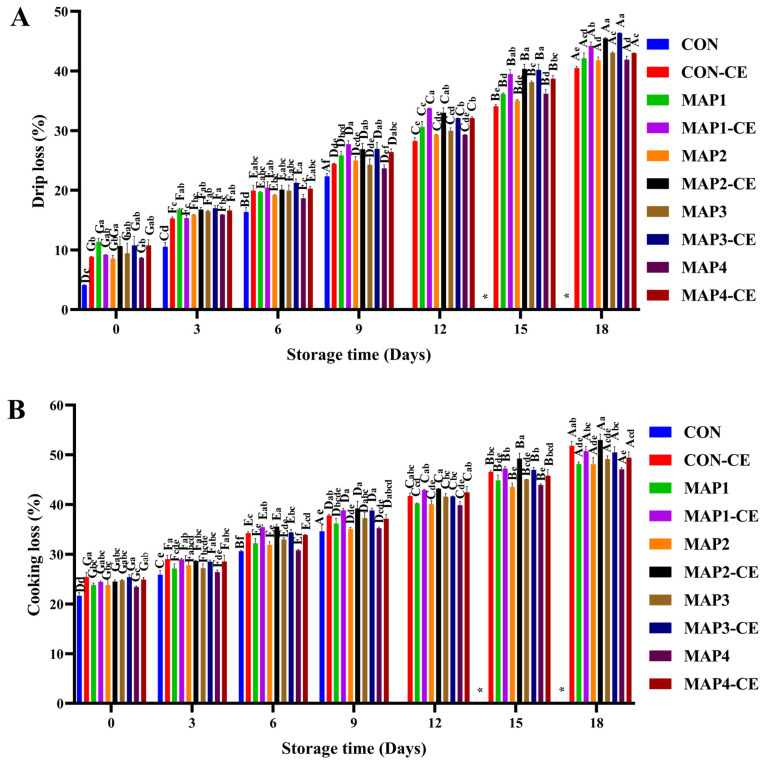

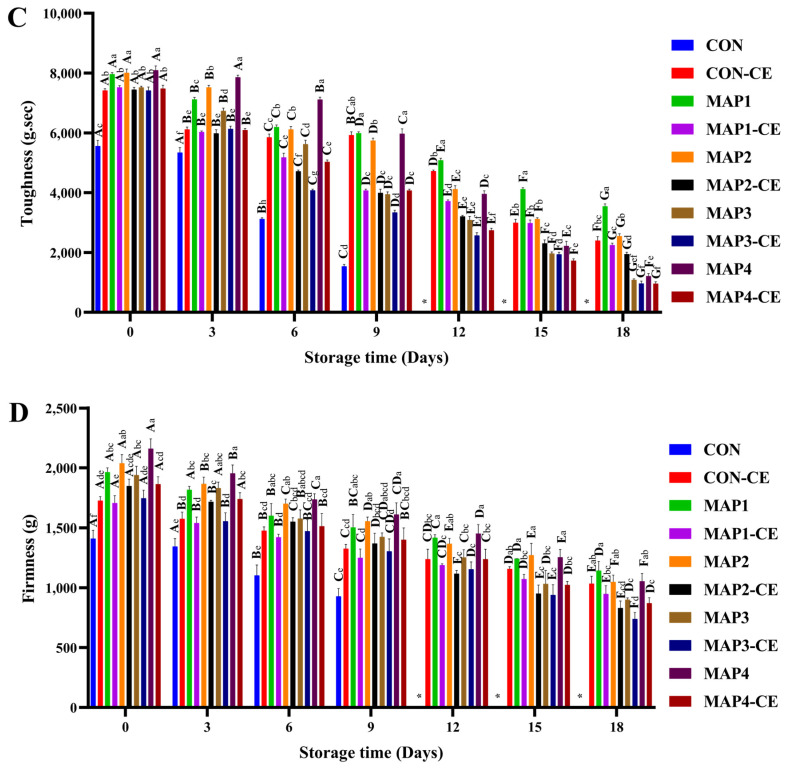
Drip loss (**A**), cooking loss (**B**), toughness (**C**), and firmness (**D**) of hard clam treated under various conditions. The error bars represent standard deviation (*n* = 3). Different lowercase letters on the bars denote significant differences between different treatments on the same storage day (*p* < 0.05). Different uppercase letters on the bars denote significant differences between the same treatments on different storage days (*p* < 0.05). *: not determined; CON: hard clam meat (HC) without any treatment; CON-CE: HC meat treated with chitooligosaccharide-epigallocatechin gallate (CE) conjugate; MAP1: HC meat packed with 40% CO_2_/20% O_2_/40% N_2_; MAP_2_: HC meat packed with 60% CO_2_/10% O_2_/30% N_2_; MAP3: HC meat packed with 80% CO_2_/20% N_2_; MAP4: HC meat packed with 80% CO_2_/20% O_2_. The samples treated with CE conjugate followed by MAP at the aforementioned gas compositions were named MAP1-CE, MAP2-CE, MAP3-CE, and MAP4-CE, respectively.

Water holding capacity (WHC) is the ability of fresh meat to retain its water, which is a quality attribute of meat products because it influences fresh weight, sensory acceptability (color, juiciness, and texture), and economic benefits. The loss of WHC in the muscle was responsible for the augmentation in drip loss and cooking loss. According to the results, MAP-treated samples, regardless of CE conjugate addition, had higher drip loss followed by CON-CE and CON, respectively (Figure 4A). Zhang et al. [40] reported higher drip loss in Golden pompano fillets when packed in MAP as compared to atmospheric packaging during super chilled storage. The drip loss continued to increase during the storage period, irrespective of sample (*p* < 0.05). The cooking loss of the samples showed a trend analogous to that of drip loss (Figure 4B). The higher drip and cooking loss in MAP-treated samples might have been due to the presence of CO_2_. When CO_2_ dissolves in the clam tissue, it forms carbonic acid, which might lower the muscle pH. Although no major changes in the pH among all samples were noticed, slightly insignificant lower pH was noticed in MAP-treated samples (Table S1). This acidification causes denaturation of myofibrillar proteins, reducing their ability to retain water. As a result, intracellular water is released, leading to greater exudate or drip loss. Moreover, MAP treatment might increase the surface hydrophobicity of HC meat through changes in protein conformation, which expose hydrophobic amino acid residues and cause a decrease in the WHC of muscle [41]. Masniyom et al. [42] reported that the exudate loss of seabass slices was approximately two times higher when 80% CO_2_ in MAP was used than when seabass slices were kept under an air atmosphere. However, drip loss did not vary with changes in gas composition, irrespective of storage day. The higher drip and cooking loss was observed when HC meat was treated with CE conjugate before MAP, irrespective of gas composition and storage day. This effect is likely due to protein aggregation induced by excessive CE conjugate, which decreases WHC and consequently increases drip loss [43]. When phenolic compounds are present in a high ratio relative to available protein sites, they tend to form a coating around protein molecules, covering their surface. This reduces protein hydrophilicity and disrupts the ability of proteins to retain water, leading to increased drip loss, as water is more easily released from the muscle [44].

The toughness and firmness of HC when subjected to MAP treatment with and without CE conjugate in comparison with CON and CON-CE are given in Figure 4C and Figure 4D, respectively. In general, firmness determines the resistance to first bite, whereas toughness measures the energy required to chew the sample. CON exhibited the lowest toughness (5560.81 g·s) and firmness (1412.20 g) when compared to treated samples on day 0 (*p* < 0.05). The higher firmness (7421.74 g) and toughness (1727.71 g·s) were obtained when HC meat was treated with CE conjugate without MAP (CON-CE) relative to CON on day 0 (*p* < 0.05). CE conjugate containing EGCG may promote protein cross-linking, which limits water mobility and improves structural integrity, resulting in higher toughness and firmness. Moreover, MAP-treated samples also exhibited higher toughness and firmness as compared to CON, regardless of gas composition on day 0. This is likely due to high CO_2_ levels in MAP, which lower tissue pH and induce protein denaturation, leading to contraction of muscle fibers. Nevertheless, lower toughness and firmness were obtained when the CE conjugate was added with MAP, irrespective of gas composition (*p* < 0.05).

Both the toughness and firmness of all samples decreased during extended refrigerated storage as compared to the sample on day 0 (*p* < 0.05). CON exhibited the highest decrease in textural properties (*p* < 0.05). This might be due to the denaturation of myofibrillar proteins, or the disruption of the connective tissues caused by proteolytic enzymes released by spoilage bacteria during storage, potentially leading to structural modifications such as the collapse and separation of muscle fibers or connective tissue in the HC meat. Moreover, there is a decrease in textural properties of HC meat packed in MAP due to the presence of CO_2_ gas, which dissolves slowly with water to form carbonic acid. The accumulation of carbonic acid led to a decrease in pH, which might cause a decrease in net charge and weaken the electrostatic repulsion.

### 3.5. Changes in Fatty Acids Composition

The fatty acids composition of samples with the lowest microbial load at the end of storage (MAP4 and MAP4-CE on day 18), their corresponding samples on day 0, as well as the CON and CON-CE on day 0 and their respective corresponding samples on the day before reaching the microbial limit of 6 log CFU/g (day 6 and day 12, respectively), are given in Table 1. Similar fatty acids were obtained in all samples on day 0 [45,46]. HC meat contains polyunsaturated fatty acids (PUFAs) such as eicosapentaenoic acid (EPA) and docosahexaenoic acid (DHA). It also consists of monounsaturated fatty acids (MUFAs) such as oleic acid, palmitoleic acid, and elaidic acid, as well as saturated fatty acids (SFAs) such as palmitic acid. In all samples, EPA (13.08 mg/100 mg lipid to 14.48 mg/100 mg lipid) and DHA (11.25 mg/100 mg lipid to 12.85 mg/100 mg lipid) were among the major PUFAs present. On day 0, the samples showed SFA content ranging from 29.86 mg/100 mg lipid to 38.04 mg/100 mg lipid, MUFA content ranging from 19.61 mg/100 mg lipid to 23.03 mg/100 mg lipid, and PUFA content ranging from 40.51 mg/100 mg lipid to 45.49 mg/100 mg lipid. In marine bivalves, PUFA content is mainly derived from their diet, which primarily consists of microalgae and phytoplankton [47]. These primary producers are rich in essential fatty acids such as EPA and DHA, which accumulate in bivalve tissues through trophic transfer. As a result, bivalves serve as valuable dietary sources of health-promoting PUFAs, enhancing the overall nutritional quality of seafood lipids. MUFA and PUFA contents were similar in CON-CE and CON on day 0, indicating that the CE conjugate had no detrimental effect on the fatty acid composition. On day 0, MAP4 and MAP4-CE showed lower levels of EPA (13.08 mg/100 mg lipid and 13.56 mg/100 mg lipid, respectively) and DHA (11.45 mg/100 mg lipid and 11.25 mg/100 mg lipid, respectively) compared to CON and CON-CE (*p* < 0.05). MUFA content also followed similar trends (*p* < 0.05). The decrease in unsaturated fatty acids might be due to O_2_, which can trigger lipid oxidation, and CO_2_, which may cause structural damage in clam tissue, increasing membrane permeability and enabling pro-oxidative enzymes such as lipoxygenase. Moreover, the addition of the CE conjugate did not influence the fatty acid composition. PUFA contents, including EPA and DHA, and MUFA contents were decreased in the CON on day 6 (*p* < 0.05). PUFA content declined to 33.72 mg/100 mg lipid, a decrease of 26% from day 0. Unsaturated fatty acids, especially PUFAs, are more susceptible to oxidation. The decline corresponded with increasing peroxides and TBARS values, indicating the primary and secondary lipid oxidation (Figure 3C,D). CON-CE also showed reduced PUFA content on day 12 as compared to day 0. However, CE conjugate slowed lipid oxidation. PUFAs dropped by 22%, and EPA and DHA were reduced to 10.43 mg/100 mg lipid and 8.02 mg/100 mg lipid, respectively. MAP-treated samples also showed a reduction in PUFA content, regardless of CE conjugate addition. MAP4 and MAP4-CE contained 38.80 mg PUFA/100 mg lipid and 40.07 mg PUFA/100 mg lipid, respectively. The former sample showed a 10% drop in PUFA level, while the latter had an 8% decline. MUFA content followed the same trend in both samples. Overall, CO_2_ in higher proportions contributed to effective microbial inhibition, thereby lowering lipid oxidation. Additionally, CE conjugate acted synergistically with MAP, further enhancing antimicrobial and antioxidant effects, thereby maintaining lipid quality.

### 3.6. Correlation Analysis Between Different Gas Compositions and Microbial/Chemical Parameters

**Figure 5:**
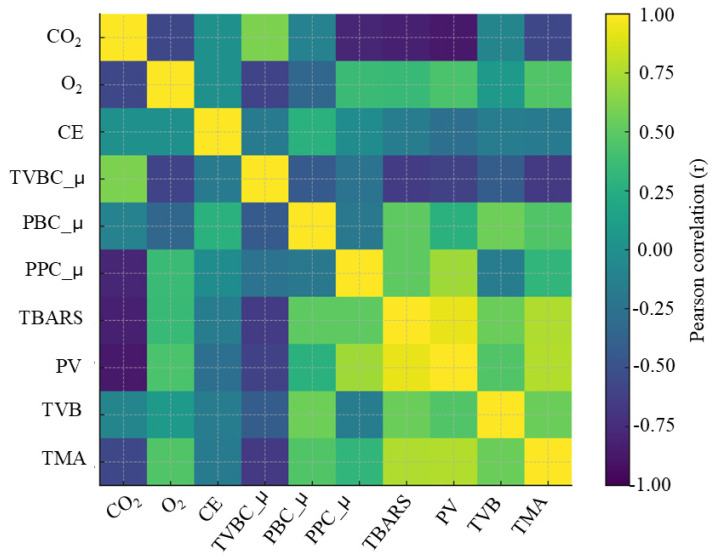
Pearson correlation of CON on day 6, CON-CE on day 15, all samples on day 18. Abbreviations: CO_2_: carbon dioxide; O_2_: oxygen; CE: chitooligosaccharide-epigallocatechin gallate conjugate; TVBC_µ: total viable bacterial count; PBC_µ: psychrophilic bacterial count; PPC_µ: Presumptive *Pseudomonas* spp. count; TBARS: thiobarbituric acid reactive substances; PV: peroxide value; TVB: total volatile bases; TMA: trimethylamine.

The correlation heat map (Figure 5) summarizes the relationships between gas composition, growth of spoilage microbiota, and chemical indices at the end of shelf-life. Strong negative correlations were observed between CO_2_ concentration and PV and TBARS content, as well as TMA-N. In contrast, O_2_ showed moderate positive correlations with the mentioned parameters, while the CE conjugate variable exhibited only weak negative associations with oxidation indices. This pattern indicates that the gas composition, particularly the balance between CO_2_ and O_2_, was the dominant driver of oxidative and nitrogenous spoilage in HC meat, whereas the CE conjugate provides only a modest additional antioxidant effect. The strong negative association between CO_2_ and the PPC_µ confirms the known bacteriostatic effect of CO_2_-enriched MAP on aerobic spoilage bacteria in fish and shellfish. High CO_2_ levels increase CO_2_ dissolution in the aqueous phase, reduce pH through carbonic acid formation, and interfere with membrane function, thereby slowing growth of *Pseudomonas* spp. and other Gram-negative spoilers. Several studies on MAP-treated salmon, tilapia, and barramundi fillets have likewise reported that atmospheres containing ≥60–75% CO_2_, combined with low O_2_, significantly suppress *Pseudomonas* proliferation and extend refrigerated shelf-life compared with air or low-CO_2_ packaging [48,49]. The positive correlation of PPC_µ with PV and TBARS content in the present data suggested that treatments permitting more rapid *Pseudomonas* growth also exhibited stronger lipid oxidation at the rejection point, consistent with the role of spoilage microflora in promoting oxidative changes via production of pro-oxidant metabolites and tissue disruption [14,50]. In parallel, the heat map shows that PV, TBARS content, and TMA-N were tightly intercorrelated, forming a distinct cluster of highly positive correlations. This indicates that primary lipid oxidation, secondary oxidation products, and TMA-N formation developed concomitantly across the gas treatments. In general, PV and TBARS determine the hydroperoxides and aldehydes from PUFA oxidation, while TMA arises from microbial reduction of trimethylamine oxide during spoilage and tends to increase alongside TVB-N and sensory deterioration in fish muscle. Coincidentally, weaker correlations were observed between gas composition and TVB-N or TMA-N than those for PV and TBARS content. MAP appears to have limited ability to suppress the formation of volatile bases once spoilage has progressed, despite clear inhibition of total microflora. This observation agrees with Debevere and Boskou [51], who showed that MAP cod fillets exhibited strongly reduced microbial counts under CO_2_-rich atmospheres, while production of TVB-N and TMA-N was only moderately affected. Overall, the heat map therefore supports the conclusion that CO_2_-rich, low-O_2_ atmospheres mainly act by slowing growth of aerobic spoilers (notably Presumptive *Pseudomonas*) and reducing lipid oxidation, while nitrogenous spoilage indices still accumulate as storage proceeds, and that the contribution of the CE conjugate, although directionally antioxidant, is secondary to the effect of gas composition.

### 3.7. Bacterial Diversity

**Figure 6:**
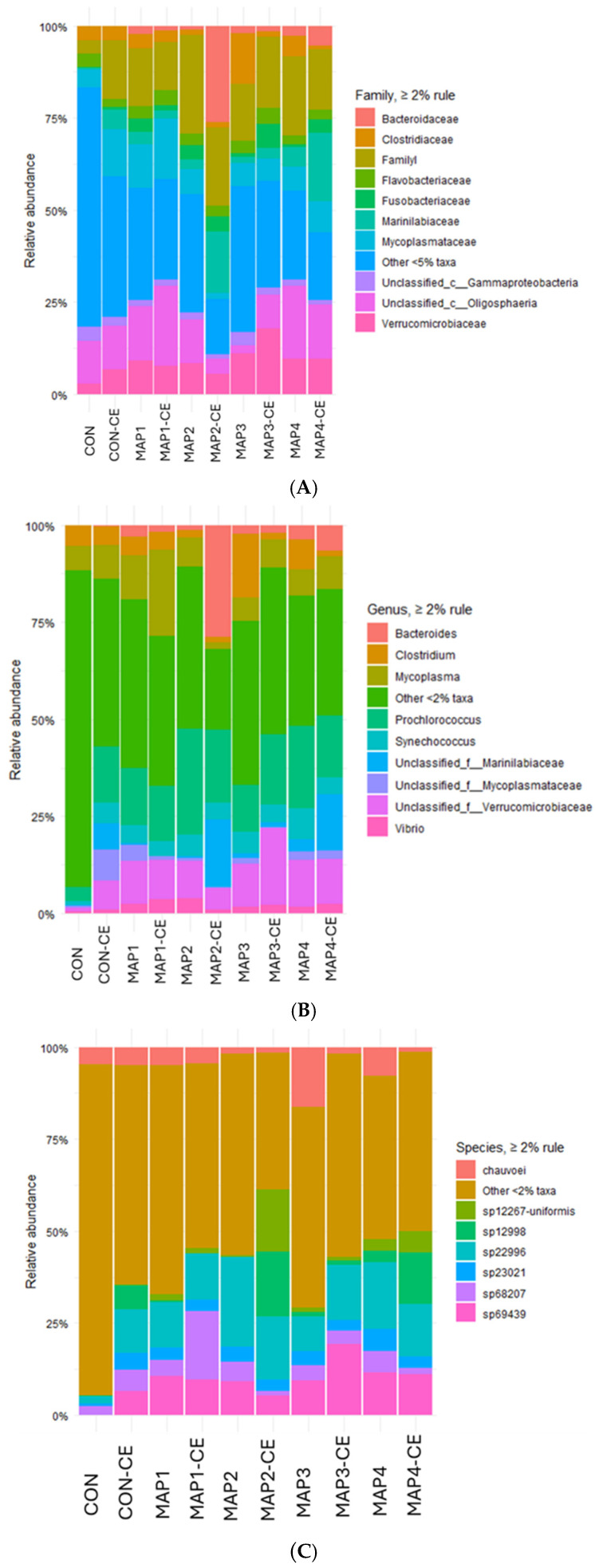
Relative abundance (%) of taxonomic groups at the family level (**A**), genus level (**B**), and species level (**C**) of CON on day 6, CON-CE on day 15, all samples on day 18. CON: hard clam meat (HC) without any treatment; CON-CE: HC meat treated with chitooligosaccharide-epigallocatechin gallate (CE) conjugate; MAP1: HC meat packed with 40% CO_2_/20% O_2_/40% N_2_; MAP_2_: HC meat packed with 60% CO_2_/10% O_2_/30% N_2_; MAP3: HC meat packed with 80% CO_2_/20% N_2_; MAP4: HC meat packed with 80% CO_2_/20% O_2_. The samples treated with CE conjugate followed by MAP at the aforementioned gas compositions were named MAP1-CE, MAP2-CE, MAP3-CE, and MAP4-CE, respectively.

The impact of MAP on bacterial diversity at the family level was compared with the CON (Figure 6A). All the samples showed a higher percentage of mixed population, as shown in ‘other’ (<5% abundance), which was the highest in CON. The result suggested that MAP and CE treatment reduced those taxa. MAP-treated samples displayed changes in the bacterial diversity as compared to CON. The relative share of unclassified Gammaproteobacteria was reduced and shifted abundance toward Flavobacteriaceae, Marinilabiaceae, and Mycoplasmataceae. The trend was consistent with the known action of CO_2_-rich atmosphere at lower temperatures. The dissolved CO_2_ diffuses across tissue, lowers intracellular pH, and inhibits key enzymes in Gram-negative seafood spoilage bacteria, thereby slowing spoilage metabolism, including amine production. CO_2_ directly suppresses the spoilage potential of *Shewanella putrefaciens* at 4 °C, and high-CO_2_ MAP broadly inhibits bacterial growth in fresh fish relative to air or vacuum [52]. The more pronounced changes were observed with the CE conjugate, aligning with reports that EGCG inhibits *Vibrio parahaemolyticus* growth and biofilm formation and that the CE conjugate acts as an enhanced antimicrobial–antioxidant system in seafood matrices; together with CO_2,_ they plausibly suppress Gammaproteobacteria and allow increases in CO_2_-tolerant families. The changes at the family level align with chemical indices (lower TMA-N and TVB-N and attenuated TBARS content and PV under high-CO_2_ MAP with CE conjugate). Overall, MAP with and without CE conjugate redirects the bacterial community rather than simply reducing alpha diversity.

The bacterial diversity on the genus level showed that MAP reshaped the microbiota toward communities dominated by *Mycoplasma* together with unclassified Marinilabiaceae and small fractions of *Bacteroides* and *Clostridium* (Figure 6B). The prominence of *Mycoplasma* under refrigerated MAP was plausible due to the lack of a cell wall and tolerance of low O_2_ and high CO_2_ conditions, while Marinilabiaceae are well-known degraders of high-molecular-weight marine substrates and frequently expand under low-O_2_, high-CO_2_ storage of seafood [53]. *Vibrio* remains present but generally at modest levels across MAP treatments, with a tendency to be lower in the CE conjugate counterparts of the same gas composition. High CO_2_ inhibits key Gram-negative aerobic spoilers via membrane diffusion, intracellular acidification, and enzyme inhibition, thereby reducing their competitive advantage at 4 °C. This mechanism is consistent with the restrained *Vibrio* in MAP-treated samples and is widely reported to underlie shelf-life extension in fish and shellfish. Where CE conjugate was present, the *Vibrio* proportion was often further suppressed relative to the MAP-treated samples with CE conjugate. CE conjugate shows enhanced inhibition of *Vibrio* and antioxidant activity suitable for seafood preservation. The low abundance of *Prochlorococcus* and *Synechococcus* represented marine cyanobacterial DNA, commonly detected in bivalve sequencing datasets, but these are not considered spoilage drivers.

**Figure 7:**
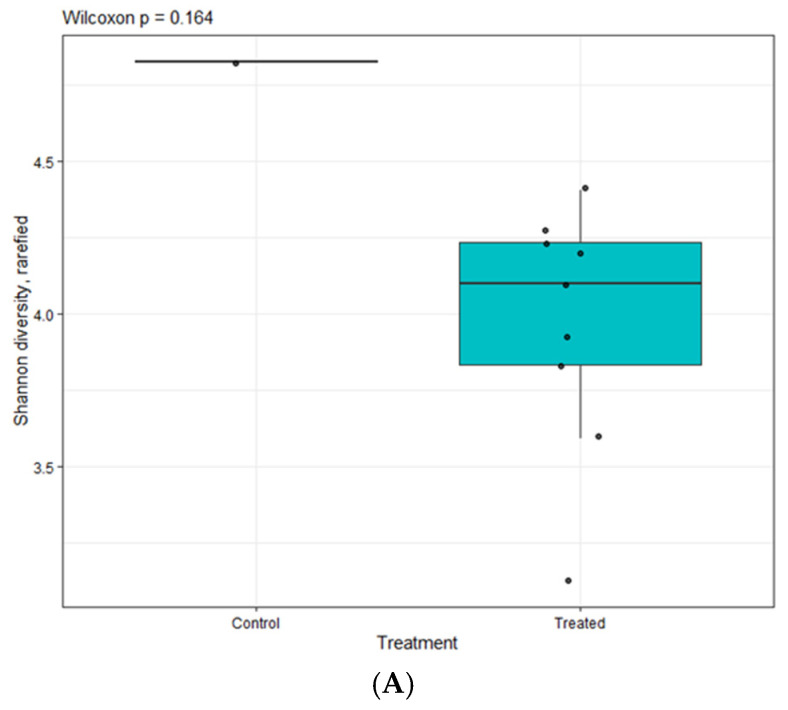

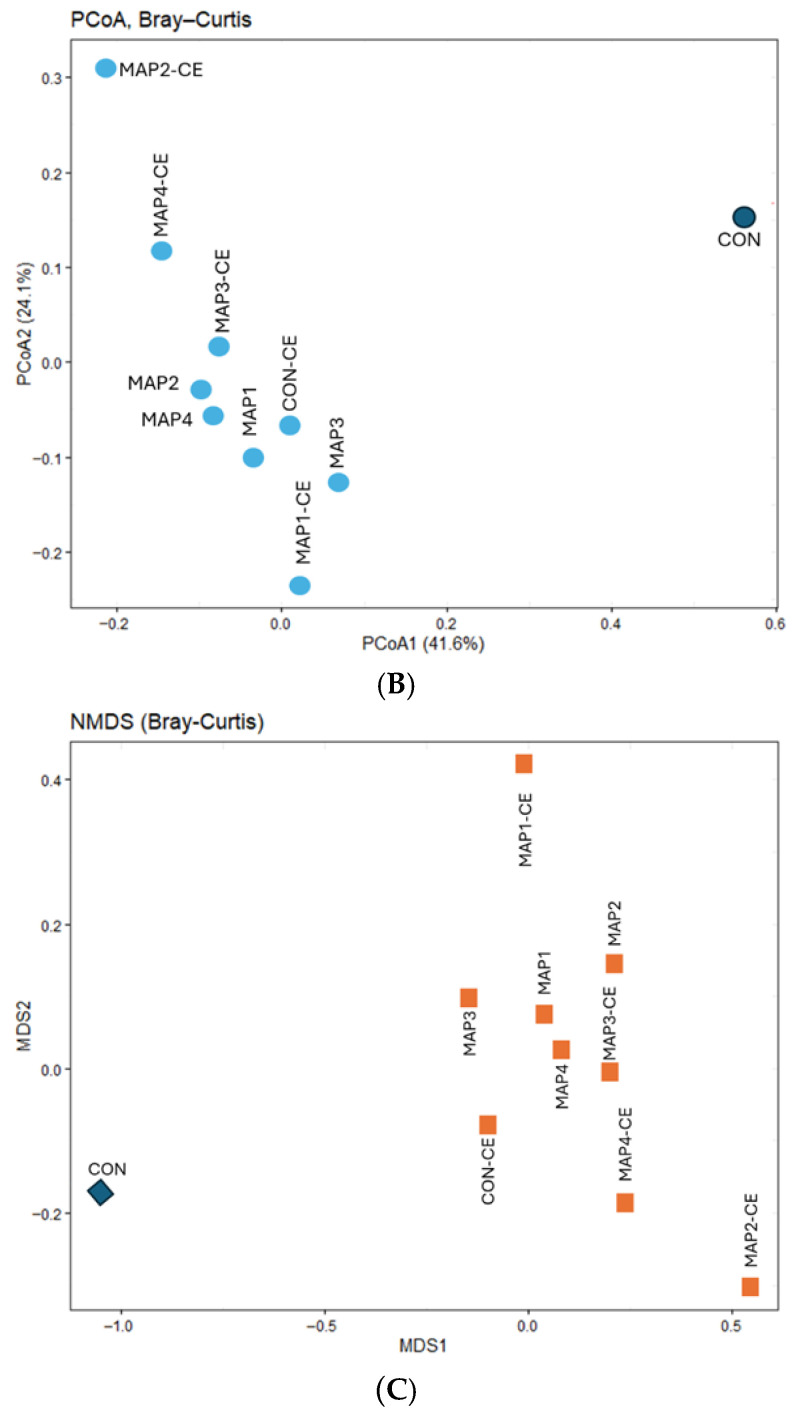
Alpha (**A**) and beta (**B**,**C**) diversity of CON on day 6, CON-CE on day 15, all samples on day 18. CON: hard clam meat (HC) without any treatment; CON-CE: HC meat treated with chitooligosaccharide-epigallocatechin gallate (CE) conjugate; MAP1: HC meat packed with 40% CO_2_/20% O_2_/40% N_2_; MAP_2_: HC meat packed with 60% CO_2_/10% O_2_/30% N_2_; MAP3: HC meat packed with 80% CO_2_/20% N_2_; MAP4: HC meat packed with 80% CO_2_/20% O_2_. The samples treated with CE conjugate followed by MAP at the aforementioned gas compositions were named MAP1-CE, MAP2-CE, MAP3-CE, and MAP4-CE, respectively.

The bacterial diversity on the species level showed that most treatments were dominated by ‘Other’ (<2% abundance), indicating a long tail of low-abundance taxa and relatively few species >2% threshold. A small abundance of species, including sp22996, sp23021, sp68207, and sp69439, contributes variably across treatments (Figure 6C). Short-read 16s rRNA sequencing of the V3–V4 region often lacks species-level resolution [54]. Therefore, species labels inferred from these amplicons should be considered putative unless validated with targeted assays or whole-genome data. MAP-treated samples displayed higher contributions from the aforementioned species than the CON. Overall, the results indicate that MAP does not yield a single dominant species but rather redistributes contributions among a small set of amplicons while maintaining a substantial sub-2% compositional shift consistent with the CO_2_-driven restraint of aerobic spoilage metabolisms in refrigerated seafood. A Wilcoxon rank-sum test was performed to compare Shannon diversity indices between CON and treated groups (Figure 7A). Shannon diversity did not differ between CON and treated samples (Wilcoxon *p* value = 0.164), indicating that within-sample richness/evenness was broadly preserved despite the compositional turnover seen in the taxa plots. The treated samples exhibited a median Shannon diversity index of approximately 4.1. The box plot indicated several data points fell both inside and outside the interquartile range (3.85–4.25), corresponding to variation in the microbial community composition among the treated samples.

The Bray–Curtis PCoA ordination showed a clear and consistent separation between CON and all MAP-treated samples (Figure 7B). CON on the far right of PCoA1, which was 41.6% of the total variation, indicated a different microbial composition compared to other samples. In contrast, MAP and MAP in combination with CE conjugate, clustered on the left half of the ordination plot. Although some vertical dispersion was observed along PCoA2 of 24.1%, the overall pattern suggested that MAP induces a major shift in microbial composition rather than minor changes in relative abundance. MAP2-CE and MAP4-CE showed slightly greater separation along PCoA2, indicating that the CE conjugate with MAP influenced the bacterial community to a higher extent. The intermediate positioning of CON-CE between CON and MAP groups suggested that CE conjugate alone influenced microbial composition, but to a lower extent as compared to MAP rich in CO_2_. Though the PERMANOVA did not reach statistical significance (*p* = 0.1012), these visual trends provide a compelling look at how the treatments transition.

Similarly, the NMDS plot (Bray–Curtis distance) showed a strong separation between the CON and all MAP-treated samples (Figure 7C). CON was on the far left along MDS1, indicating a distinct microbial community during refrigerated storage of HC meat. In contrast, all MAP and MAP-combined CE conjugate treatments shifted to the right side of the ordination. The results indicated a clear shift in microbial composition. All treated samples formed a compact cluster and spread moderately along both MDS1 and MDS2, suggesting that MAP consistently changes microbial composition regardless of specific gas composition or CE conjugate addition. CON-CE was positioned between CON and MAP-treated samples, suggesting that CE conjugate alone influenced microbial community, but not to the extent relative to MAP with CO_2_ at higher proportions. Notably, MAP in combination with CE conjugate samples occupied ordination space similar to that of their MAP-only counterparts, implying that CE conjugate provides an additive but smaller directional shift in community structure. NMDS stress value (0.044) indicated a good representation of the dissimilarity data in two dimensions. Supporting the PERMANOVA findings, the ANOSIM also showed no significant differences between groups (R = 0.9938, *p* = 0.1013). Overall, MAP was the primary factor for shifting microbial composition in HC meat, while CE conjugate exhibited an additive effect, slightly modulating bacterial composition.

## 4. Conclusions

Hard clam (HC) meat could be stored in atmospheric air for up to 9 days based on viable bacterial counts, whereas pretreatment with the CE conjugate followed by MAP extended its microbiological shelf-life to 15–18 days, depending on gas composition. HC meat stored under 80% CO_2_, particularly in MAP4 and MAP4-CE, exhibited lower microbial load, reduced volatile base content, and less lipid oxidation, albeit at the cost of higher drip and cooking losses. Further studies should evaluate the use of water-holding additives such as hydrocolloids or protein-based binders to mitigate exudate formation during MAP without compromising safety and product quality. High-throughput 16S rRNA sequencing further revealed that MAP reshaped the bacterial community towards CO_2_-tolerant taxa while maintaining overall alpha diversity, with clear separation between CON and MAP (±CE) in beta-diversity ordinations. Correlation analysis supported the dominance of CO_2_ concentration in bacterial growth and spoilage chemistry, while CE conjugate providing a secondary but beneficial antimicrobial–antioxidant effect. This work was limited to one bivalve species, a single storage temperature (4 °C), and one CE conjugate dose, and did not include detailed sensory or consumer evaluations. Future studies should optimize CE conjugate concentration and gas ratios, explore WHC-enhancing ingredients to reduce drip loss, integrate sensory/consumer acceptance, and combine metabarcoding with culture-dependent or metagenomic approaches. Furthermore, determination of protein structural changes in protein that underline the increased drip and cooking losses observed in MAP-treated HC meat are also warranted.

## Figures and Tables

**Table 1:** Fatty acids composition of hard clam meat treated without and with chitooligosaccharide-epigallocatechin (CE) conjugate with modified atmospheric packaging (MAP) while stored at 4 °C.

Fatty Acids (mg/100 mg Lipid)	Day 0				Day 6	Day 12	Day 18	
	CON	CON-CE	MAP4	MAP4-CE	CON	CON-CE	MAP4	MAP4-CE
C14:0 Myristic	3.04 ± 0.14 ^bB^	3.12 ± 0.03 ^bA^	2.69 ± 0.04 ^cA^	3.25 ± 0.03 ^aA^	3.65 ± 0.22 ^A^	1.94 ± 0.03 ^B^	1.65 ± 0.02 ^bB^	1.83 ± 0.11 ^aB^
C14:1 Myristoleic	1.36 ± 0.07 ^aA^	1.21 ± 0.00 ^cA^	1.02 ± 0.02 ^dA^	1.24 ± 0.01 ^bA^	0.83 ± 0.02 ^B^	ND	ND	ND
C16:0 Palmitic	24.35 ± 0.62 ^aA^	22.33 ± 0.15 ^bA^	22.35 ± 0.39 ^bA^	24.55 ± 0.30 ^aA^	22.90 ± 0.43 ^B^	18.09 ± 0.09 ^B^	14.75 ± 0.83 ^bB^	16.40 ± 0.11 ^aB^
C16:1 Palmitoleic	8.52 ± 0.21 ^cA^	8.74 ± 0.04 ^bA^	8.25 ± 0.14 ^cA^	9.38 ± 0.07 ^aA^	7.14 ± 0.12 ^B^	5.76 ± 0.16 ^B^	4.72 ± 0.08 ^bB^	5.15 ± 0.25 ^aB^
C17:0 Heptadecanoic acid	1.54 ± 0.11 ^aA^	1.44 ± 0.16 ^aA^	1.23 ± 0.17 ^bB^	1.03 ± 0.11 ^bB^	1.62 ± 0.18 ^A^	1.06 ± 0.73 ^A^	4.31 ± 0.61 ^aA^	5.90 ± 1.83 ^aA^
C17:1 Heptadecenoic acid	1.74 ± 0.06 ^aA^	1.52 ± 0.02 ^bA^	ND	0.48 ± 0.00 ^cA^	0.63 ± 0.01 ^B^	ND	ND	ND
C18:0 Stearic	1.01 ± 0.11 ^cA^	1.22 ± 0.22 ^cA^	9.07 ± 0.20 ^aB^	7.91 ± 0.10 ^bB^	1.10 ± 0.01 ^bA^	1.27 ± 0.08 ^aA^	10.89 ± 0.08 ^aA^	11.03 ± 0.59 ^aA^
C18:1 Elaidic	5.05 ± 0.22 ^aB^	4.99 ± 0.07 ^aB^	4.30 ± 0.10 ^bB^	4.15 ± 0.04 ^bB^	6.62 ± 0.06 ^A^	3.95 ± 0.05 ^A^	8.58 ± 0.06 ^aA^	8.19 ± 0.49 ^aA^
C18:1 Oleic	3.36 ± 0.11 ^aB^	3.13 ± 0.01 ^bB^	2.97 ± 0.06 ^cA^	3.24 ± 0.02 ^aA^	3.75 ± 0.12 ^A^	2.26 ± 0.05 ^A^	2.55 ± 0.04 ^aB^	2.44 ± 0.12 ^aB^
C18:2 Linoleic	0.82 ± 0.02 ^aB^	0.31 ± 0.27 ^dB^	0.49 ± 0.02 ^cB^	0.58 ± 0.02 ^bA^	0.94 ± 0.04 ^A^	1.43 ± 0.19 ^A^	1.12 ± 0.08 ^aA^	ND
C18:3 Linolenic	4.09 ± 0.26 ^bA^	6.65 ± 0.05 ^aA^	4.62 ± 1.33 ^bB^	2.71 ± 0.49 ^cB^	2.82 ± 0.08 ^B^	5.31 ± 0.06 ^B^	7.80 ± 0.26 ^aA^	5.07 ± 1.31 ^bA^
C20:1 Eicosenoic	2.75 ± 0.06 ^cA^	3.44 ± 0.02 ^aB^	3.08 ± 0.34 ^abB^	2.97 ± 0.34 ^bcA^	1.39 ± 0.02 ^B^	4.07 ± 0.02 ^A^	3.75 ± 0.05 ^aA^	3.29 ± 0.22 ^bA^
C20:2 Eicosadienic	4.80 ± 0.02 ^aA^	4.70 ± 0.13 ^aA^	4.36 ± 0.04 ^bA^	4.41 ± 0.11 ^bA^	4.12 ± 0.02 ^B^	4.48 ± 0.04 ^B^	3.86 ± 0.04 ^aB^	3.62 ± 0.19 ^bB^
C20:3 Eicosatrienoic	0.65 ± 0.05 ^bA^	0.62 ± 0.01 ^bB^	0.82 ± 0.07 ^aB^	0.45 ± 0.04 ^cB^	0.69 ± 0.02 ^A^	0.92 ± 0.01 ^A^	0.99 ± 0.04 ^aA^	0.89 ± 0.05 ^bA^
C20:4 Eicosatetraenoic	7.81 ± 0.26 ^bcA^	7.92 ± 0.09 ^bA^	8.50 ± 0.19 ^aB^	7.53 ± 0.07 ^cB^	5.60 ± 0.33 ^B^	5.93 ± 0.10 ^B^	8.71 ± 0.11 ^aA^	6.29 ± 0.60 ^aA^
C23:0 Tricosanoic	1.80 ± 0.11 ^aA^	1.75 ± 0.03 ^aB^	1.74 ± 0.05 ^aB^	1.30 ± 0.03 ^bB^	3.65 ± 0.06 ^B^	3.10 ± 0.06 ^A^	3.01 ± 0.05 ^aA^	2.71 ± 0.16 ^bA^
C20:5 Eicosapentaenoic	14.48 ± 0.12 ^aA^	14.32 ± 0.04 ^aA^	13.08 ± 0.25 ^cA^	13.56 ± 0.11 ^bA^	11.15 ± 0.78 ^B^	8.02 ± 0.04 ^B^	6.70 ± 0.06 ^aB^	10.79 ± 0.38 ^aB^
C22:6 Docosahexaenoic	12.85 ± 0.25 ^aA^	12.59 ± 0.10 ^aA^	11.45 ± 0.24 ^bB^	11.25 ± 0.13 ^bB^	9.40 ± 0.21 ^B^	10.43 ± 0.33 ^B^	9.64 ± 0.19 ^aA^	10.40 ± 0.85 ^aA^
Saturated fatty acids	31.73 ± 0.60 ^bB^	29.86 ± 0.20 ^cB^	37.08 ± 0.75 ^aA^	38.04 ± 0.39 ^aA^	32.92 ± 0.65 ^cA^	25.46 ± 0.54 ^A^	34.60 ± 0.36 ^bB^	37.87 ± 0.94 ^aA^
Monounsaturated fatty acids	22.77 ± 0.09 ^bA^	23.03 ± 0.07 ^aA^	19.61 ± 0.05 ^dA^	21.46 ± 0.21 ^cA^	22.36 ± 0.20 ^aB^	15.03 ± 0.17 ^aB^	19.60 ± 0.06 ^cA^	19.06 ± 0.15 ^bB^
Polyunsaturated fatty acids	45.49 ± 0.67 ^bA^	47.11 ± 0.16 ^aA^	43.31 ± 0.72 ^cB^	40.51 ± 0.21 ^dB^	33.72 ± 0.74 ^bB^	36.51 ± 0.71 ^aA^	38.80 ± 0.36 ^aA^	37.07 ± 0.82 ^bA^

The results are presented as means ± SD (*n* = 3). Different lowercase letters in the same row denote significant differences between different treatments on the same storage day (*p* < 0.05). Different uppercase letters in the same row denote significant differences between the same treatments on different storage days (*p* < 0.05). ND: not detected; CON: hard clam meat (HC) without any treat-ment; CON-CE: HC meat treated with chitooligosaccharide-epigallocatechin gallate (CE) conjugate; MAP4: HC meat packed with 80% CO_2_/20% O_2_; MAP4-CE: HC meat packed treated with CE conjugate followed by packed with 80% CO_2_/20% O_2_.

## Data Availability

The original contributions presented in this study are included in the article/Supplementary Material. Further inquiries can be directed at the corresponding author.

## Supplementary Materials

The following supporting information can be downloaded at: https://www.mdpi.com/article/10.3390/foods15061026/s1, Table S1: pH of HC meat under different treatments during refrigerated storage.
