# Supplementary material for: Combined Effects of Gas Composition in Modified Atmosphere Packaging and Chitooligosaccharide-EGCG on Quality Changes in Refrigerated Asian Hard Clam Meat
Source: Foods. 2026 Mar 15;15(6):1026. doi: 10.3390/foods15061026 (PMC13025460; doi:10.3390/foods15061026)
Supplement: Supplementary file 1 [file foods-15-01026-s001.zip › foods-4182393-supplementary.pdf]

Table S1. pH of HC meat under different treatments during refrigerated storage.

Treatment	Day 0	Day 3	Day 6	Day 9	Day 12	Day 15	Day 18
**CON**	6.46±0.02^{a}	6.56±0.04^{a}	6.79±0.02^{b}	6.62±0.01^{c}	–	–	–
**CON-CE**	6.89±0.01^{b}	6.87±0.01^{b}	6.64±0.01^{a}	6.27±0.01^{a}	6.46±0.02^{a}	6.92±0.02^{a}	6.62±0.01^{a}
**MAP1**	7.43±0.01^{c}	7.02±0.01^{c}	7.27±0.03^{d}	6.46±0.02^{b}	6.41±0.01^{a}	7.26±0.01^{d}	7.09±0.01^{c}
**MAP1-CE**	7.49±0.01^{c}	7.35±0.01^{e}	7.25±0.01^{d}	7.28±0.02^{h}	7.17±0.01^{f}	7.14±0.01^{c}	7.12±0.01^{c}
**MAP2**	7.53±0.04^{d}	7.38±0.02^{e}	7.06±0.04^{c}	6.86±0.02^{d}	6.89±0.03^{c}	7.15±0.01^{c}	7.13±0.02^{d}
**MAP2-CE**	7.64±0.04^{e}	7.22±0.01^{d}	7.21±0.02^{d}	6.95±0.01^{e}	6.96±0.01^{d}	7.10±0.01^{c}	6.97±0.01^{b}
**MAP3**	7.46±0.01^{c}	7.04±0.04^{c}	7.26±0.04^{d}	7.26±0.00^{h}	7.30±0.01^{g}	7.30±0.02^{d}	7.08±0.01^{c}
**MAP3-CE**	7.56±0.04^{d}	7.29±0.01^{d}	7.21±0.05^{d}	7.19±0.02^{g}	7.09±0.01^{e}	7.01±0.01^{b}	7.10±0.02^{c}
**MAP4**	7.38±0.05^{c}	7.30±0.01^{d}	7.07±0.01^{c}	7.04±0.02^{f}	6.68±0.02^{b}	6.92±0.01^{a}	7.00±0.01^{b}
**MAP4-CE**	7.58±0.01^{d}	7.25±0.01^{d}	7.07±0.02^{c}	6.79±0.01^{d}	6.87±0.01^{c}	7.05±0.01^{b}	7.09±0.00^{c}

Values are mean ± SD (n = 3).

Different superscript letters within the same column indicate significant differences ($p < 0.05$).

"–" indicates that the sample was not analysed at that time point (CON was not followed beyond day 9 due to microbiological spoilage).
